# Predicting asymptomatic neurosyphilis using peripheral blood indicators

**DOI:** 10.1186/s12879-021-06846-6

**Published:** 2021-11-26

**Authors:** Weijie Li, Jiaqi Han, Pan Zhao, Dagang Wang, Tianhao Sun, Jie Guo, Yanqun He, Pei Qu, Ying Liu, Congle Shen, Yajie Wang

**Affiliations:** 1 Department of Clinical Laboratory, Beijing Ditan Hospital Capital Medical University, Beijing, China; 2 ICU, The First Hospital of Tsinghua University, Beijing, China; 3 Department of Infectious Diseases, The Fifth Medical Center, Chinese PLA General Hospital, Beijing, China; 4 Department of Orthopaedics and Traumatology, The University of Hong Kong-Shenzhen Hospital, Shenzhen, China

**Keywords:** Asymptomatic neurosyphilis, Neurosyphilis, Lumbar puncture, Cerebrospinal fluid, Logistic regression model

## Abstract

**Background:**

The high misdiagnosis rate of asymptomatic neurosyphilis (ANS) has long challenged infectious disease clinicians. We aim to develop a model for diagnosing ANS in asymptomatic syphilis (AS) patients without CSF indicators.

**Results:**

277 AS patients with HIV-negative and underwent lumbar puncture were enrolled in this horizontal study.The area under the curve for predicting ANS by CSF leukocytes and protein was 0.643 and 0.675 [95% CI, 0.583–0.699VS.0.616–0.729]. Through LRM, the AUC increased to 0.806 [95% CI, 0.732–0.832], and the Youden's index was 0.430. If the score is ≤ 0.159, ANS can be excluded with a predictive value of 92.9%; we can identify ANS while the score is over 0.819, with a predictive value of 91.7% and a specificity of 99.25%. This study showed that the LRM can diagnose ANS in AS patients effectively.

**Conclusion:**

Given a large number of misdiagnosis ANS patients and CSF results' insufficiency, the model is more practical. Our research will help clinicians track suspected syphilis, especially those who cannot accept the CSF test.

## Background

Syphilis, asymptomatic or symptomatic, is caused by *Treponema pallidum* infection and is a serious global health issue [1–4]. Treponema invades the nervous system in about one-third of patients beginning on days following primary infection, and then proceed into neurosyphilis (NS) [5]. Subsequent NS may be classified as asymptomatic neurosyphilis or symptomatic neurosyphilis and as early (1–2 years after primary infection) or late [6]. Although NS occurs at any stage of syphilis and has various clinical manifestations, ANS is the most common [7]. About 13.5% of latent syphilis patients had ANS, which were more likely to have late neurological complications[8].

Confirmation of ANS requires evidence of *Treponema pallidum* invading the central nervous system. That is challenging and contentious. Lumbar puncture (LP) performing is necessary for CSF examination, but its invasive nature makes it unacceptable for most patients, especially those who had AS [9, 10]. Whether AS patients need CSF examination has always been debatable; there is insufficient evidence to suggest that the identification of asymptomatic neurosyphilis helps to predict treatment outcomes, even in patients with HIV [11]. According to the report, only 35% of asymptomatic patients with HIV have accepted recommended CSF examination in practice [12]. Despite all that, the ANS diagnosis most depends on the combination of abnormal results of serum and CSF syphilis tests and elevation in the CSF white-cell count and protein content so far, but no consensual definition exists. These tests are imperfect and have no benchmarks [6]. WHO [13] and the Centers for Disease Control and Prevention (CDC, USA) recommend CSF-VDRL as the choice method for the laboratory diagnosis of neurosyphilis [14], but the sensitivity of test for neurosyphilis is low, only about 30 to 70% [15–17]. Polymerase chain reaction (PCR) testing is the preferred method, but it is insensitive in using CSF or blood (the sensitivity varied between 40 and 70%) [18–23]. There is no internationally approved PCR assay for *Treponema pallidum*. These, together with its asymptomatic or protean clinical manifestations which mimic other diseases, lead to the diagnosis tending to be overlooked [24–27]. In fact, although the suspect has undergone routine CSF examinations, clinicians are increasingly diagnosing and tracking suspicious patients; And since CSF assessment is not without its dangers, it is not recommended for the vast majority of asymptomatic patients by the latest guideline [17]. The Canadian Public Health Laboratory Network recommends a serodiagnosis of syphilis as an entry point into the diagnostic algorithm for suspected neurosyphilis, but treponemal-specific markers may sometimes be indeterminate [28].

We performed this study to analyse the results of syphilis-related indicators in serum and CSF of AS patients, establish a model to predict the possibility of ANS, and test it.

## Methods

### Patients

Each participant underwent clinical evaluation, including medical history, physical and neurological examinations, and laboratory testing. Enrollment Eligibility included clinical and serological evidence of symptomatic syphilis.

We collected CSF and serum simultaneously for the toluidine red unheated serum test (TRUST), *treponema pallidum* particle agglutination (TPPA), fluorescent TP absorption test (FTA-ABS), routine examinations besides CSF biochemical, PBTS test.

Diagnostic criteria for asymptomatic non-neurosyphilis (ACS): (i) No clinical manifestations of syphilis. (ii) Serological *Treponema pallidum* particle TPPA or TRUST positive, LP to exclude NS. (iii) A history of syphilis, blood transfusion, or occupational exposure of the suspects or his mother.

ANS diagnosis refers to the 2015 UK national guidelines on the management of syphilis [29], no nervous system symptoms involvement. The serum and CSF meet the followings: (i) Serological test for syphilis and the TRUST of CSF (c-TRUST) is positive. (ii) If c-TRUST is negative, the TPPA of both serology (s-TPPA) and CSF (c-TPPA) is positive; white blood cells count (c-WBC) over 5 copies/μL or protein concentration exceeds (c-Pro) 45 mg/L in CSF. No other aetiology causes such elevation.

### Laboratory methods

We purchased the TPPA reagents from Japan's Fuji Rubber Co., Ltd., FTA-ABS (FTA-ABS-IgG, FTA-ABS-IgM) reagents from Germany Oumeng Medical Laboratory Diagnostics Co., Ltd. TRUST agents were purchased from Shanghai Rongsheng Biotech Company. Peripheral blood lymphocyte subsets (CD3^+^, CD3^+^CD4^+^, CD3^+^CD8^+^, CD45^+^) analysis was performed on a FACSCalibur flow cytometer, reagents were also provided by BD Company (BD Biosciences, San Jose, CA). We carried the peripheral blood and CSF routine examinations out on the Sysmex Xe.4000 blood cell analyser.

### Statistical analyses

Results are expressed as the mean ± standard deviation or median (range or interquartile) where appropriate. We compared categorical data between groups with the corrected chi-square or two-sided Fisher exact test, compared parametric quantitative data using the Student's t or one-way analysis and non-parametric data through the Mann‐Whitney test, analysing the relationship by logistic regression analysis. We assessed the accuracy of parameters in differentiating ANS from ACS patients using the area under the receiver operating characteristic (ROC) curve. We considered it statistically significant at a p-value < 0.05, performed statistical analyses by the SPSS statistical software (version 30.0, SPSS Inc).

## Results

### Population

From January 2014 to September 2019, we enrolled 277 AS cases from Beijing Ditan Hospital Capital Medical University in this cross-sectional study. All cases, including 128 males and 149 females, underwent lumbar puncture with an average age of 41 years (28.3–54.8 years). The characteristics of participant patients are shown in Table 1.

**Table 1:** Baseline characteristics of patients enrolled in the trial

Parameters	Total(277)	ANS(143)	ACS(134)	*P*-value (Two-tailed)
Sex(male/female)	128/149	(78/65)	(50/84)	0.004*
Age (years)	41.59 ± 13.26	45(35–54)	35(27.75–47)	< 0.001#
CSF-T	61.31 ± 290.06	14(7–31)	7(5–12)	< 0.001#
CSF-WBC	7.88 ± 25.90	5(3–10)	3(2–4)	< 0.001#
CSF-Pro	31.1 ± 14.91	32.3(24.1–42)	25(17.93–34.05)	< 0.001#
CSF-G	3.62 ± 0.69	3.46(3.24–3.71)	3.56(3.27–3.84)	0.212#
CSF-Cl	125.72 ± 4.32	126.08 ± 2.2	125.75(124.48–127.2)	0.179#

When we grouped subjects, the WBC and protein in CSF were different, so we did not do the statistical analysis

*CSF-T* total number of cells in CSF, *CSF-WBC* white blood cells in CSF, *CSF-Pro* protein in CSF, *CSF-G* glucose in CSF, *CSF-Cl* chloride in CSF

Statistical analysis used in this table: *Independent sample t-test, two-tailed; #Non-parametric Mann–Whitney U-test

In the ANS group, there were 143 cases (52%), including 78 males and 65 females. The average age was 45 years (35–54 years), older than the ACS Group. Among them, 48 cases (33.57%) showed elevated WBC, 20 cases (13.99%) showed enhanced protein and 11 cases (7.69%) with elevated CSF protein and WBC.

There were 134 patients (48%) with ACS, with an average age of 35 years (27.75–47 years). Among them, WBC increased in 17 cases (12.69%), protein increased in 8 cases (5.97%), and 2 cases (1.49%) of elevated WBC and protein coexisted (Table 1). It lacked differences in glucose and chloride levels between the two groups.

### Immunological characteristics and lymphocyte subsets of ANS and ACS groups

In the AS patients, the seropositivity rate of syphilis non-specific (TRUST) and specific test (TPPA) exceeded that of CSF, 90.91% vs. 8.30% and 98.92% vs. 57.03%, *P* < 0.001.

The seropositivity rate of TRUST in the ANS group was 90.91%, with the average titer 17.82 (1–128); when coming to that of CSF, it was 0.33. The TPPA positive rate of serum and CSF was 99.31% vs.81.81%.

**Fig. 1:**
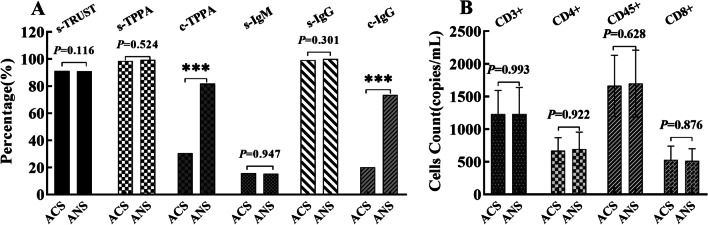
Immunology and lymphocyte subsets of patients with ANS (left) and ACS (right). **A** Comparison of immunological characteristics on ANS and ACS group. **B** Analogy of lymphocyte subsets results of ANS and ACS group

In the ACS group, the seropositivity rate of TRUST was 91.05%, and the average titer was 7.53, but the results of the c-TRUST examination were negative. The TPPA positive rate of serum and CSF was 98.51% VS. 30.60%. Both the TRUST and TPPA positive rate of CSF in the ANS group was higher than those in the ACS group (*P* = 0.001), but not the seropositivity rate of TRUST and TPPA (*P* = 0.116, *P* = 0.524). Still, the average s-TRUST titer of the ANS group was higher than that of the ACS group (*P* = 0.004), and the trend was the same in CSF (*P* < 0.001). The positive rate of IgG in CSF of the ANS group was higher than that of the ACS group (*P* = 0.001), while there was no significant diversity in the seropositive rates of IgG and IgM (*P* = 0.301 VS. *P* = 0.947), as showing in Fig. 1A.

The invasion of syphilis to the nervous system related to the cellular and humoral immunity of subjects [30], and T, B, and NK cells played a decisive role in the regulation. To explore the clinical value, we analysed the lymphocyte subsets of ANS. The results revealed that there was not any difference in inhibition/toxicity, auxiliary/induction and natural killer cell count between the two groups (*P* = 0.922,* P* = 0.628,* P* = 0.876, Fig. 1B). Results showed that the immunity imbalance of syphilis was not significant between the two groups.

### Peripheral blood routine examinations in ANS and ACS patients

**Fig. 2:**
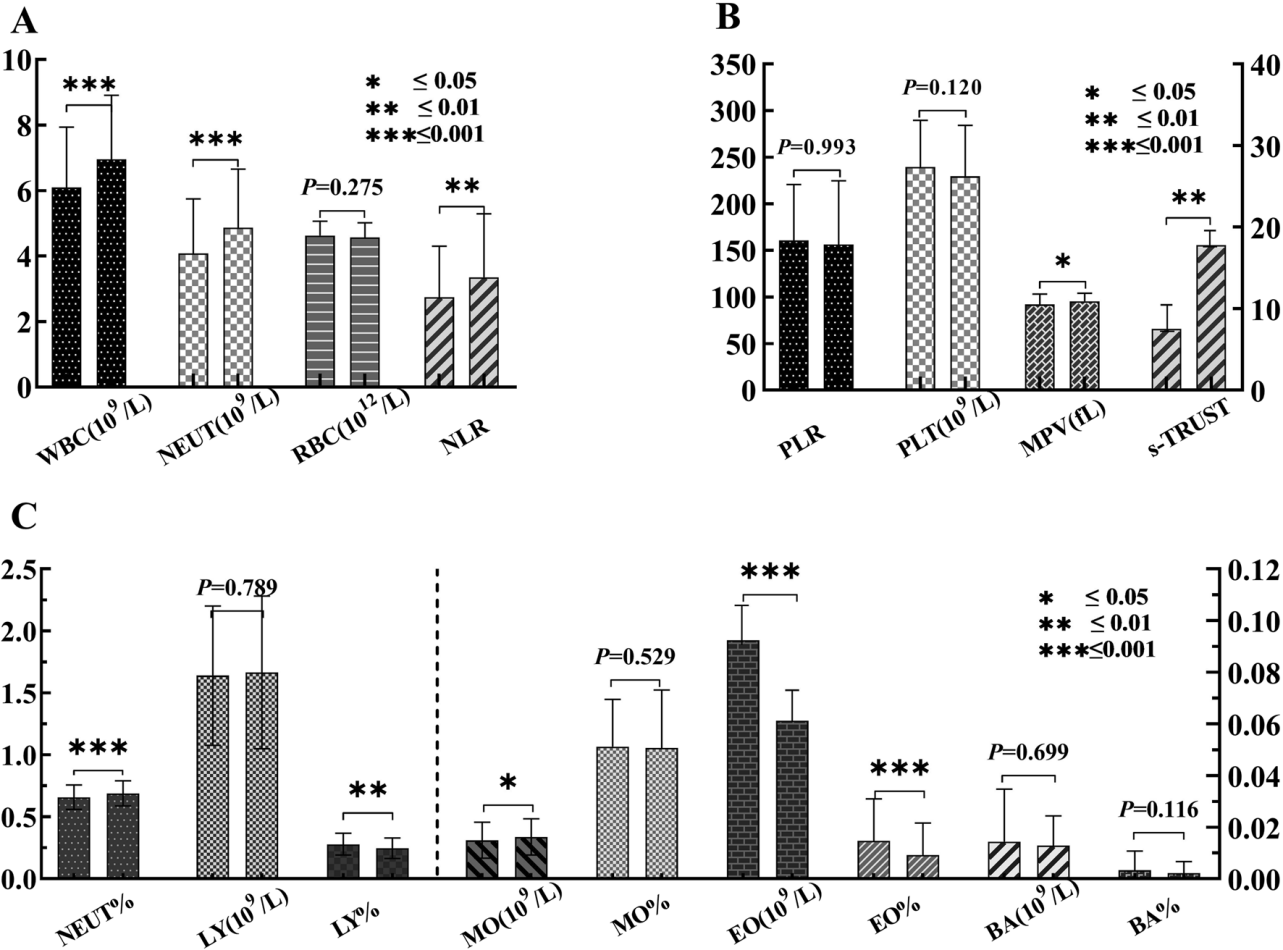
Blood routine results of ANS (left) and ACS (right) patients. **A** The comparison between leukocytes and neutrophils. **B** Comparison of MPV and s-TRUST titers. *C* comparison of LY, EO, MO

Neutrophils and lymphocytes engaged in the inflammatory response of NS. At present, the ratio of neutrophil to lymphocyte (NLR) is a new indicator of systemic inflammation, which can further reflect the body's inflammatory state, an NLR of 1.65 might be a reference for healthy people [31, 32]. Our results showed that the NLR of the ANS and ACS groups was 2.91 (1.96–4.16) VS. 2.39 (1.81–3.1), *P* = 0.002. Ratios of platelets to lymphocyte (PLR) in peripheral blood were 0.14 (0.11–0.19) and 0.15 (0.12–0.19), respectively, with no significant difference (*P* = 0.993). The examination of peripheral blood showed the major differences between them were leukocytes and platelets, but not red blood cells (Fig. 2).

### Relationship of age, s-TRUST titer, and ANS

**Fig. 3:**
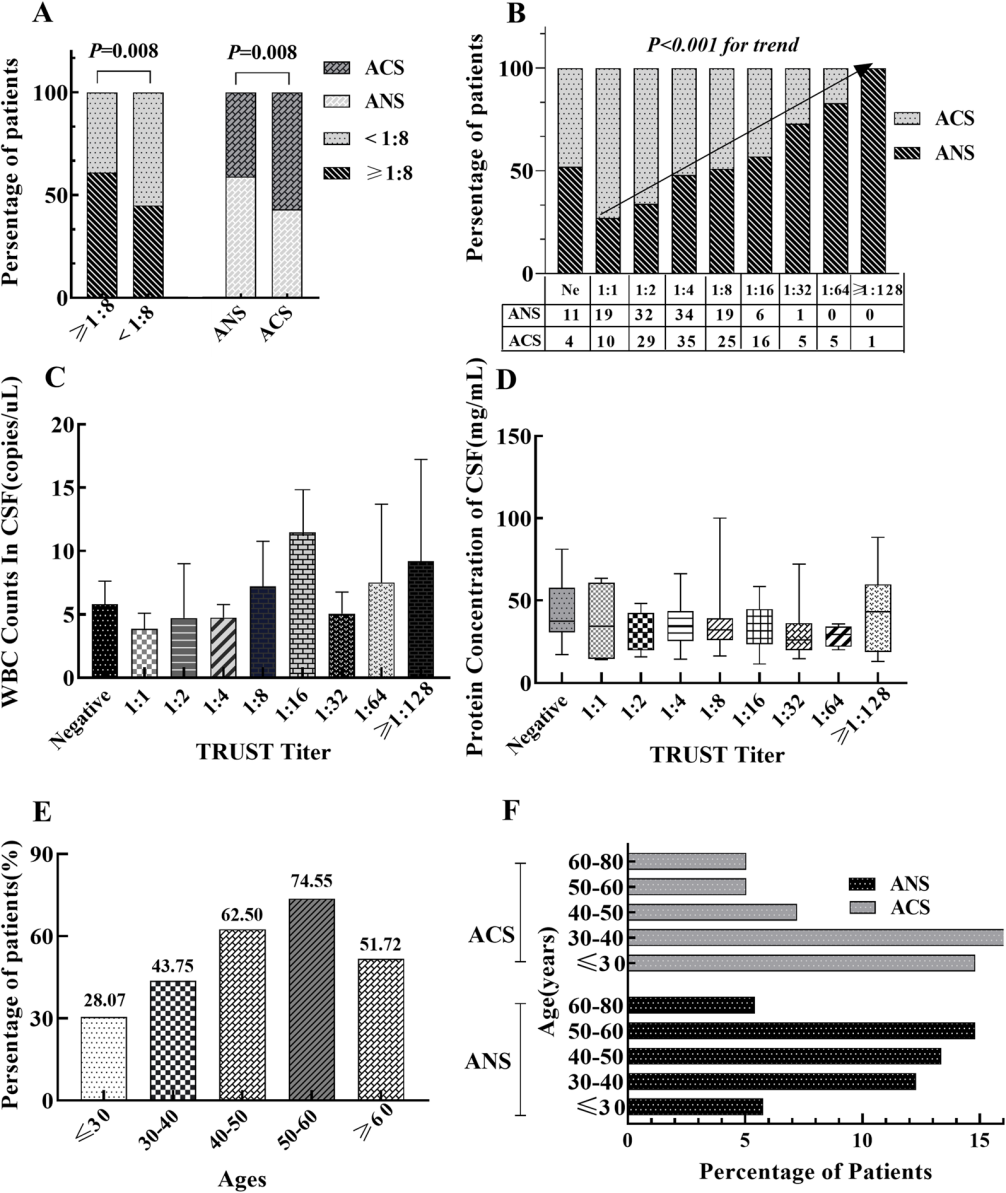
Relationship between age, s-TRUST titer and ANS. **A** The proportion of patients in TRUST titer greater than or less than 1:8 groups. **B** The proportion of patients at different TRUST levels. **C** White blood cell counts of CSF in patients with different TRUST titers. **D** CSF protein concentration in different TRUST titer groups. **E** The proportion of ANS patients among AS patients of different ages. **F** Relation of age between ANS and ACS patients

The area under the ROC curve showed that s-TRUST titer and age were the best predictors at the critical value of 1: 8 and 39 years, Youden's indexes were 0.292 VS. 0.170, sensitivity was 65.50% VS. 63.6%, and the specificity was 64.18% VS. 80.60%. The proportion of patients with TRUST titer ≥ 1:8 in the ANS group was higher than that in the ACS group [60.84% (87/143) VS. 44.78% (60/134)], *P* = 0.008. In cases with TRUST titer ≥ 1:8, the percentage of ANS exceeded that with TRUST titer < 1:8, 43.08% (56/130) vs. 59.18% (87/147), *P* = 0.008 (Fig. 3A). Among patients with positive TRUST result, the TRUST titer correlated with ANS positively, as shown in Fig. 3B (r = 0.269, *P* < 0.001). When the titer of TRUST was 1: 128, the proportion of ANS in AS patients reached 100%, although the number is minimal. However, the c-WBC count or protein content did not elevate with the increase of TRUST titer, as shown in Fig. 3C, D. In AS patients, the occurrence of ANS shows an increasing trend with age (r = 0.233, *P* = 0.001). The occurrence of ANS in the 50–60-year-old group exceeded the 20–30-year-old group (74.55% (41/55) vs. 28.07% (16/57), *P* = 0.001). However, in cases over 60 years, the ANS incidence did not increase but decrease (Fig. 3E). 88.81% of ANS patients were over 30 years, predominantly 50–60 years old; while 69.40% of the ACS group were older than 30 years, primarily 30–40 years (Fig. 3F).

### ANS diagnoses in AS based on laboratory parameters

**Fig. 4:**
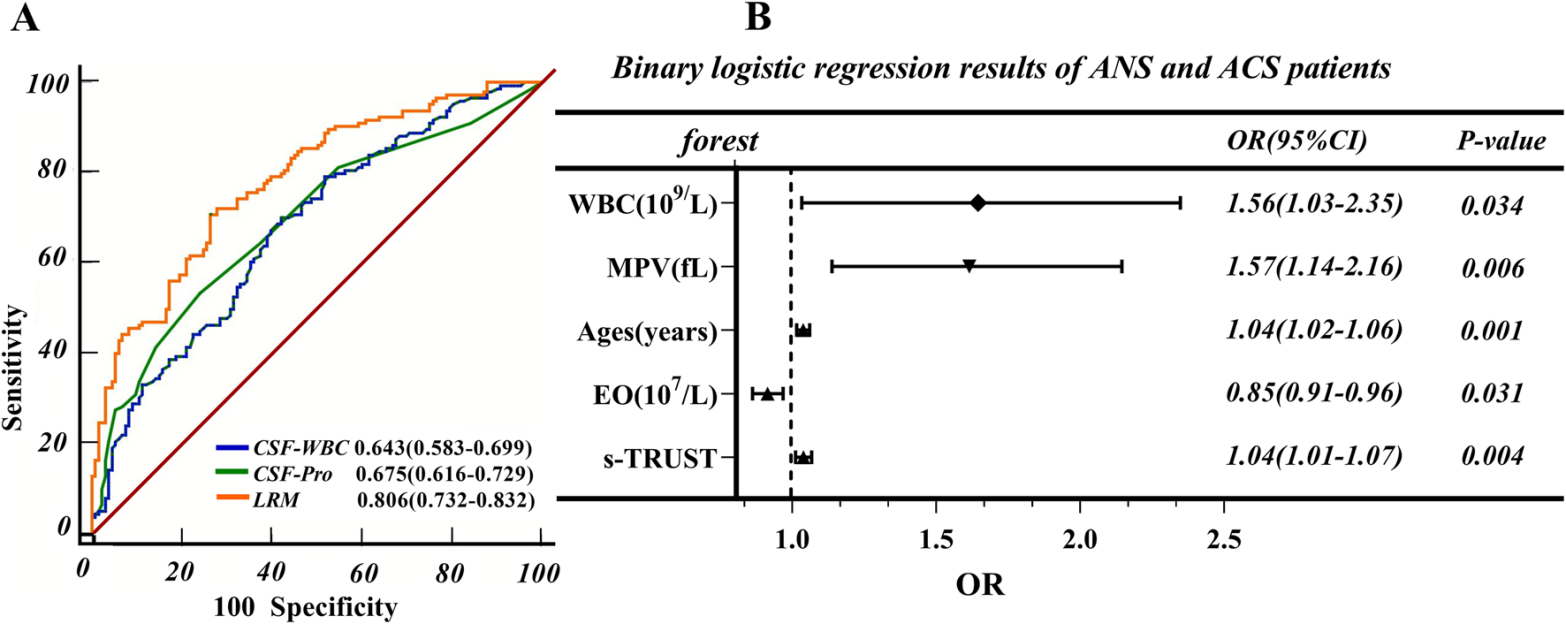
Analysis of LRM. **A** Comparison of c-WBC, c-Pro and LRM by the ROC area. **B** LRM forest map

Correlation analysis showed that age (r = 0.233, *P* < 0.001), gender (r = 0.173, *P* = 0.004), WBC (r = 0.202, *P* < 0.001), NEUT/LY (r = 0.184, *P* = 0.002), NEUT (r = 0.215, *P* < 0.001), NEUT% (r = 0.145, *P* = 0.003), LY% (r = − 0.153, *P* = 0.002), MO (r = 0.105, *P* = 0.034), EO (r = − 0.197, *P* < 0.001), EO% (r = − 0.211, *P* < 0.001), MPV (r = 0.126, *P* = 0.011), serum TRUST titer (r = 0.206, *P* = 0.001) associated with ANS. In order to establish the diagnostic model for ANS in AS patients, collinearity diagnostic was performed on the mentioned indicators that are meaningful and differentiated. Results demonstrated that WBC, NEUT, NEUT%, EO, EO% have multicollinearity (Variance Inflation Factor > 5). After principal component analysis, dimensionality reduction processing, then all indicators were included in the bivariate regression analysis. The results show that age (*P* = 0.001, Exp [B] = 0.037), MPV (*P* = 0.006, Exp [B] = 0.451), s-TRUST (*P* = 0.038, Exp [B] = 1.039), EO (*P* = 0.002, Exp [B] =  − 9.871), and WBC (*P* = 0.034, Exp [B] = 0.443) associated to the occurrence of ANS independently. Subsequently, we established the following LRM based on the above parameters: Y = 0. 037 × age + 0. 451 × MPV + 0. 038 × s-TRUST-9. 871 × EO + 0. 443 × WBC, the goodness-of-fit test of logistic regression model by Hosmer–Lemeshow shows that the model has a good fit with the observed values, *P* = 0.581. A forest plot was then produced to offer a visualization of the relationship among the ANS risk-related factors identified in the previous group analysis (Fig. 4B). The incidence of ANS increased by 3.78%, when the age increased by 1 year. The MPV increased by 1 fL, WBC increased by 1 × 10^9^/uL, and the probability of ANS increased by 56.98% and 55.74% respectively. But the increase in EO counts means a decrease in the likelihood of ANS.

The AUC of LRM to predict ANS occurrence was 0.806 [95% CI, 0.732–0.832] (Fig. 4A). The cutoff value for predicting ANS was 0.503, and the Youden's index was 0.430, with a diagnostic sensitivity of 70.63% and a specificity of 72.39%. With the cutoff value ≥ 0.819 in LRM, the positive predictive value is 91.7%, with a specificity of 99.25%, showing a higher predictive power than the protein concentration or WBC count in CSF. With a cutoff value at 0.159 in LRM, we can exclude the diagnosis of ANS with a predictive value of 92.90% and a sensitivity of 99.30%, Fig. 4.

## Discussion

*Treponema pallidum* remains one of the human pathogens that cannot be cultivated in vitro to-date. Suitable animal models for the pathogenesis of it also lacking [17]. These obstacles have hindered the effort of elucidating the basic immunological traits and diagnosis of syphilis infection. There is still no "gold standard" for diagnosing currently. Some patients refused to seek medical attention and reject LP because of shameful and complications after the LP procedure (such as headache, infection, and bleeding). In addition, the inconsistency of the results with NS clinical manifestation sometimes, further contributing to misdiagnosis. The above reasons drove us to establish a more sensitive, noninvasive, easy-to-accept and effective diagnostic model.

ANS can be manifest as abnormalities in peripheral blood and CSF. To establish this model, we comprehensively analysed the results of syphilis-related items in the patient' serum and CSF. The analysis showed WBC, NEUT/LY, NEUT, LY%, MO, EO, MPV, TRUST, age, and gender-related to ANS. Based on age, MPV, s-TRUST, EO and WBC, which included in the above factors, LRM was estimated. We can diagnose ANS in AS patients effectively by it. The AUC for the ANS diagnosis by serum LRM is 0.806, and the cutoff value is 0.503, with Youden's index 0.430. It recommends that patients with LRM ≥ 0.819 should undergo an LP to rule out ANS as soon as possible. Given the limitations of CSF examination, clinicians should consider empirical nervous system treatment regardless of the results. If serum LRM ≤ 0.159 and no neurological symptoms or signs, we do not recommend performing LP for these patients.

In our study, the serum TRUST titer of ANS exceeded that of the ACS patients. Although 13 of 143 patients with ANS had negative TRUST results, the elevation in TRUST titer associated with ANS. That consistent with the previous report (which showed that serum RPR titer correlated with NS) positively [5, 33, 34]. However, those studies have not analysed NS typing separately. This research showed the negative result of TRUST in AS patients cannot rule out ANS. There was no statistical difference in TRUST positive rate between the ACS and ANS groups, but the percentage of ANS patient in the 1:1, 1:2, 1:4, 1:8, and 1:16 groups gradually increased. Our result indicated that predicting ANS in AS by TRUST titer alone is challenging.

WBC count and protein concentration of CSF is the most used for syphilis nervous system infection. Similar to previous studies [17], our results show that CSF abnormalities related to ANS, but up to 40% of the cases, one or both of them are normal, indicating that they contain higher specificity rather than sensitivity [24, 33]. At the cutoff value of 24 cells/μL, c-WBC counts were helping for diagnosing ANS in AS patients, with a PPV of 83.3%, but the sensitivity is only 3.5%. For limitations on CSF examinations, we recommend that clinicians consider empiric treatment for patients with a high LMR score, regardless of whether it is in the normal range.

Despite the large sample size, this research is restricted by its single-centre, retrospective design. In fact, we did not dynamically monitor changes and could not determine the syphilis staging of all events, although repeated syphilis was more likely to be asymptomatic [35]. Future research needs to conduct multi-centre studies and dynamically observe the differences before and after ANS to explore diagnostic indicators' value.

In summary, the long-term consequences of untreated ANS are high morbidity and mortality. Considering the limitations of CSF results and LP risks, it will benefit from using the convenient LMR in patients diagnosed with AS. This basis considers empirical therapy for patients with a high pre-test probability of neurosyphilis without routine CSF analysis.

## Data Availability

The datasets analysed during the current study are available from the corresponding author on reasonable request.

## Abbreviations

ANS: Asymptomatic neurosyphilis
LP: Lumbar puncture
CSF: Cerebrospinal fluid
LRM: Logistic regression model
AS: Asymptomatic syphilis
TRUST: Toluidine red unheated serum test
TPPA: *treponema pallidum* Particle agglutination test
FTA-ABS: Fluorescent TP absorption test
